# ORF1a of highly pathogenic PRRS attenuated vaccine virus plays a key role in neutralizing antibody induction in piglets and virus neutralization in vitro

**DOI:** 10.1186/s12985-017-0825-2

**Published:** 2017-08-22

**Authors:** Chaoliang Leng, Wuchao Zhang, Hongliang Zhang, Yunchao Kan, Lunguang Yao, Hongyue Zhai, Mingliang Li, Zhen Li, Chunxiao Liu, Tongqing An, Jinmei Peng, Qian Wang, Yumin Leng, Xuehui Cai, Zhijun Tian, Guangzhi Tong

**Affiliations:** 1 State Key Laboratory of Veterinary Biotechnology, Harbin Veterinary Research Institute, Chinese Academy of Agricultural Sciences, No. 427, Maduan Street, Nangang District, Harbin, 150001 China; 2 Henan Key Laboratory of Insect Biology in Funiu Mountain, Henan Provincial Engineering Laboratory of Insects Bio-reactor, China-UK-NYNU-RRes Joint Laboratory of Insect Biology, Nanyang Normal University, Nanyang, 473061 China; 3 College of Physics and Electronic Engineering, Nanyang Normal University, Nanyang, 473061 China; 4 Shanghai Veterinary Research Institute, Chinese Academy of Agricultural Sciences, No. 518, Ziyue Road, Minhang District, Shanghai, 200241 China

**Keywords:** PRRSV, Chimeric virus, Neutralizing antibody, Neutralization region

## Abstract

**Background:**

Currently, porcine reproductive and respiratory syndrome virus (PRRSV) is one of the most economically important viral pathogens in swine in most countries, especially China. Two PRRSV attenuated live vaccine strains (HuN4-F112 and CH-1R) are currently widely used in China. Our previous study showed that HuN4-F112, but not CH-1R, induced high anti-nucleocapsid (N) antibody and neutralizing antibody (NA) titers. Additionally, sera from HuN4-F112 inoculated pigs induced low cross neutralization of CH-1R.

**Methods:**

In the present study, 6 chimeric viruses through exchanging 5′ untranslated region (UTR) + open reading frame (ORF)1a, ORF1b, and ORF2–7 + 3’UTR between HuN4-F112 and CH-1R were constructed and rescued based on the infectious clones of rHuN4-F112 and rCH-1R. The characteristics of these viruses were investigated in vitro and vivo.

**Results:**

All the three fragments, 5’UTR + ORF1a, ORF1b, and ORF2–7 + 3’UTR, could affect the replication efficiencies of rHuN4-F112 and rCH-1R in vitro. Additionally, both 5’UTR + ORF1a and ORF2–7 + 3’UTR affected the anti-N antibody and NA responses targeting rHuN4-F112 and rCH-1R in piglets.

**Conclusions:**

The 5’UTR + ORF1a region of HuN4-F112 played a key role in inducing NAs in piglets. Furthermore, we confirmed for the first time that ORF1a contains a neutralization region. This study provides important information that can be used for further study of the generation of anti-PRRSV NAs.

## Background

Currently, porcine reproductive and respiratory syndrome (PRRS) is one of the most economically important diseases affecting the swine industry worldwide [1, 2]. The causative agent of this disease is the PRRS virus (PRRSV), which causes reproductive failure in sows and respiratory disease in pigs of all ages. This virus emerged in North America and central Europe in the late 1980s [3–5].

PRRSV, belonging to the family *Arteriviridae* in the order *Nidovirales*, is an enveloped single-stranded positive-sense RNA virus [6, 7]. Its genome is approximately 15 kb and consists of a 5’untranslated region (UTR), at least ten overlapping open reading frames (ORFs), and a 3’UTR [8]. Once the genome enters the cytoplasm, ORF1a and ORF1b are translated to produce two large polyproteins (pp1a and pp1ab) [9]. pp1ab expression is controlled by a − 1 ribosomal frameshift [10]. Autocatalytic processing of these precursors generates at least 14 non-structural proteins (NSPs) that are involved in virus replication and transcription [11, 12]. ORF1a encodes 10 NSPs (NSP1α, NSP1β, NSP2–6, NSP7α, NSP7β and NSP8), and ORF1b encodes 4 NSPs (NSP9–12) [13–15]. Recently, an additional viral protein synthesized by a − 2 ribosomal frame shift in the NSP2 coding region has been described [16]. ORF2a, ORF2b and ORFs 3–7 encode the viral structural proteins GP2, E, GP3, GP4, GP5, M and N, respectively [8]. Additionally, a novel ORF overlapping the GP5 coding sequence has been discovered [17, 18].

PRRSV is divided into two major genotypes as follows: genotype 1 (European type, EU-type) and genotype 2 (North American type, NA-type) [19]. The representative strains of the two genotypes are Lelystad virus (LV) and VR-2332, respectively. These viruses share only approximately 70% nucleotide sequence identity [3, 4]. In 2006, an unparalleled, large-scale, atypical PRRS outbreak caused by a highly pathogenic PRRSV (HP-PRRSV) was reported in China [20, 21]. Currently, HP-PRRSV is recognized as the dominant virus in China and southeast Asian countries [22]. Phylogenetic analyses showed that both genotype 1 and 2 PRRSVs could be divided into three subtypes [23, 24]. Additionally, there have been frequent reports of a variety of atypical PRRSV strains in China recently [24–26].

To control this severe disease, many PRRS attenuated live vaccines have been prepared. Among these vaccines, HuN4-F112 and CH-1R have been widely used in China. These strains were obtained by culture of the parental strains HP-PRRSV HuN4 and classical PRRSV CH-1a and passaged on MARC-145 cells for 112 and 165 passages, respectively [27, 28]. Our laboratory found that HuN4-F112 but not CH-1R induced high neutralizing antibody (NA) and N antibody titers in piglets [29]. Currently, the generation of anti-PRRSV NAs is poorly understood. A characteristic feature of PRRSV infection is that anti-PRRSV NAs appear late after infection, whereas natural infection or vaccination induces only low NA levels [7, 30]. Additionally, despite the attempts of many laboratories, a neutralizing monoclonal antibody against PRRSV has not been developed. Furthermore, our previous study found that the B epitope, which is the major neutralizing epitope of classical PRRSV, was not a neutralizing epitope in HP-PRRSV [29, 31]. Thus, exploration of the generation of anti-PRRSV NAs and the identification of the protective neutralizing antigenic region of PRRSV for potential use for the development of a vaccine are very useful and encouraging approaches.

In this study, we used the infectious clones (rHuN4-F112 and rCH-1R) of attenuated HP-PRRSV vaccine strain HuN4-F112 and classical PRRSV vaccine strain CH-1R as backbones and constructed a series of chimeric clones by individually exchanging the corresponding regions within the genome between the two parental clones. Then, we rescued the viruses and analyzed the growth kinetics, the ability to induce antibodies and NAs, and the viremia in piglets. Additionally, significant differences in cross NA titers of anti-rHuN4-F112 sera against the different rescued viruses were analyzed.

## Methods

### Viruses and cells

The attenuated live HP-PRRSV vaccine strain HuN4-F112 and classical PRRSV vaccine strain CH-1R (GenBank accession: EU807840) were obtained by culturing the parent strain HP-PRRSV HuN4 (GenBank accession: EF635006) and the classical strain PRRSV CH-1a (GenBank accession: AY032626) on MARC-145 cells [27, 28]. BHK-21 cells were used to rescue the virus by transfection within vitro transcribed RNA. MARC-145 cells were used for virus rescue and the subsequent experiments. The two cell lines were cultured in Dulbecco’s modified Eagle’s medium (DMEM) (Invitrogen) supplemented with 10% fetal bovine serum (FBS) (HyClone) at 37 °C in a humid atmosphere with 5% CO_2_.

### Construction of PRRSV chimeric full-length cDNA clones

**Fig. 1:**
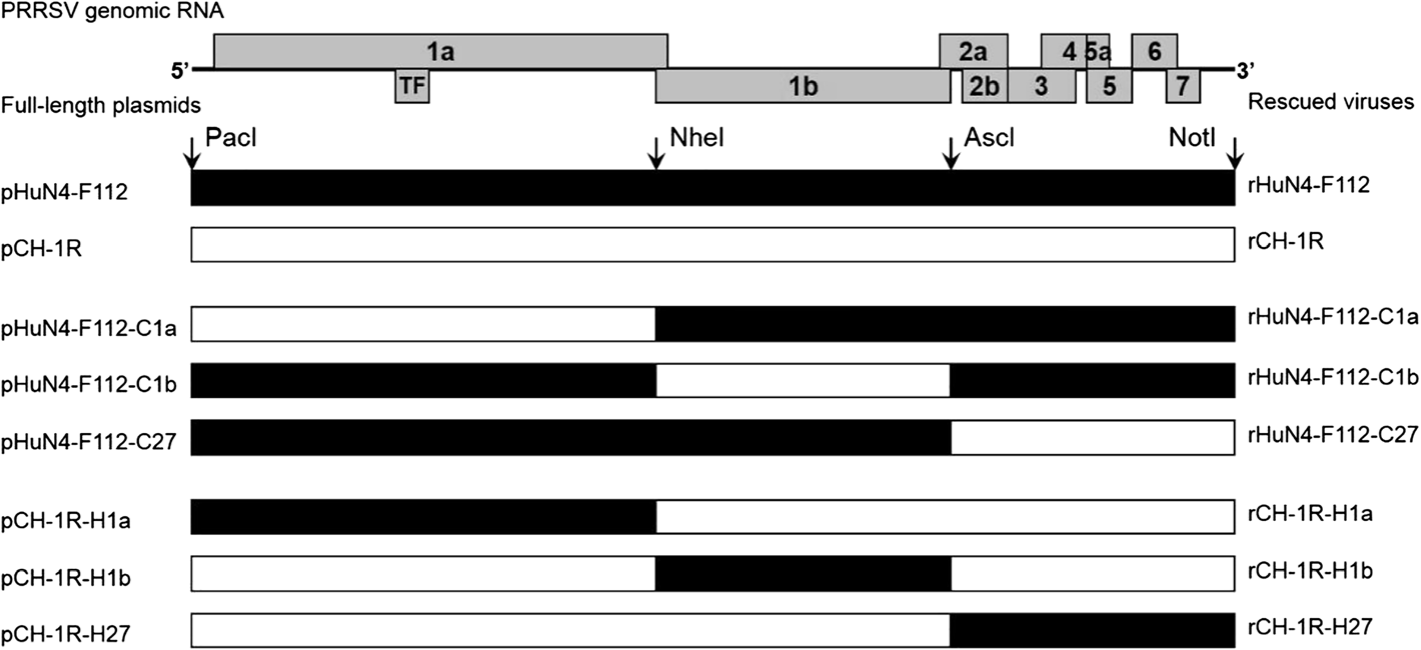
Construction strategy for the full-length PRRSV chimeric cDNA clones. The full-length chimeric infectious clones were constructed by exchanging 5’UTR + ORF1a, ORF1b, and ORF2–7 + 3’UTR between HuN4-F112 and CH-1R. The boxes represent the genomic fragments of the parental backbone viruses rHuN4-F112 (black) and rCH-1R (white). The unique restriction enzyme sites used for cloning are shown above the bars. The designation of each full-length plasmid is shown on the left side, and each rescued virus is shown on the right side

The PRRSV full-length infectious cDNA clone plasmids pHuN4-F112 and pCH-1R were previously constructed and identified by our laboratory [32, 33]. The strategy used for the construction of PRRSV chimeric full-length cDNA clones is illustrated in Fig. 1. The 5’UTR + ORF1a, ORF1b, or ORF2–7 + 3’UTR region was swapped between pHuN4-F112 and pCH-1R using the unique restriction enzymes (New England Biolabs) *Pac*I and *Nhe*I, *Nhe*I and *Asc*I, or *Asc*I and *Not*I, respectively. The corresponding fragments were connected using the T4 DNA ligase (TaKaRa). The chimeric full-length cDNA clones with the pHuN4-F112 backbone were designated pHuN4-F112-C1a, pHuN4-F112-C1b and pHuN4-F112-C27. Correspondingly, the chimeric clones with the pCH-1R backbone were named pCH-1R-H1a, pCH-1R-H1b and pCH-1R-H27.

### Recovery of the chimeric and parental viruses

The protocol for the rescue of the chimeric and parental viruses was performed as previously described [32]. First, each full-length cDNA clone plasmid was separately linearized using the restriction enzyme *Not*I (New England Biolabs). The linearized plasmid DNA was transcribed and capped using the mMessage High Yield Capped RNA Transcription kit (Ambion) according to the manufacturer’s instructions. The synthetic RNA was transfected into BHK-21 cells using the DMRIE-C reagent (Invitrogen) according to the manufacturer’s protocol. Finally, the supernatants were harvested 24 h post-transfection and serially passaged on MARC-145 cells.

### Identification of the rescued viruses

The rescued viruses were examined by cytopathic effects (CPE) and indirect immunofluorescence assay (IFA). Briefly, the rescued virus suspensions were inoculated onto MARC-145 cell monolayers prepared in 6 well plates for 24 h (h) earlier. And then the cells were incubated at 37 °C and monitored daily for CPE. The IFA protocol was described previously [34]. Antigens of each rescued virus were prepared by inoculating MARC-145 cell monolayers in alternate rows of 96-well plates with 100 μL/well of the virus at the titer of 10^4^ 50% tissue culture infective doses per mL (TCID_50_/mL) and fixing infected cells with cold acetone-ethanol (8:2) mixture after a 20 h incubation at 37 °C. Uninfected cell monolayers used as cell control antigens were prepared in the identical manner using virus-free cell culture medium. All plates were dried and stored at −20 °C until use. The presence of PRRS viral antigen in each plate was confirmed by immunofluorescence microscopy using PRRSV specific monoclonal antibody 1C8 [35] and goat anti-mouse IgG antibody conjugated with FITC (Sigma) as the primary and second antibodies, respectively.

To further confirm the rescued PRRSVs, the complete genomes of the second-passage viruses were sequenced. The RT-PCR primers and protocols were previously described [24]. Briefly, the genomic RNAs were extracted using an RNA extraction QIAamp viral RNA Mini Kit (Qiagen) and RT-PCR was carried out with the One Step RT-PCR kit (Qiagen) according to the manufacturer’s instructions. The amplified PCR products were subjected to agarose gel electrophoresis and the target fragments were excised from the gels for purification at a later stage, which was performed using a Gel Extraction Kit (OMEGA, USA). The purified PCR products were cloned into a pMD18-T vector (TaKaRa, Japan). Recombinant clones were sent to Life Technologies (Shanghai, China) for sequencing. To further assess the replication stability, the rescued viruses were serially passaged on MARC-145 cells for 10 generations. And the complete genomes of the rescued PRRSVs of generations 5 and 10 were also sequenced.

### Growth kinetics of the rescued viruses

To analyze the growth kinetics in vitro, MARC-145 cell monolayers in 6-well plates were individually infected with each chimeric virus and their parental viruses at the same multiplicity of infection (MOI) of 0.1. The viral titers in the supernatants at different time points were determined using the Reed-Mench method [36] and expressed as TCID_50_/mL. Briefly, MARC-145 cell monolayers were prepared in 96-well plates and inoculated with virus suspensions (100 μL/well) prepared by serial 10-fold dilution. Each diluted sample was run in eight parallel repeats in 96-well plates. And the plates were incubated for an additional 72 to 96 h. The viral titers were determined by the presence of visible CPE. The experiment was independently repeated three times and each time point was also independently repeated three times. The results represent the average of the duplicates.

### Animal trials of the rescued viruses

Thirty-three 28-day-old PRRSV-free Bama piglets were obtained from the Laboratory Animal Center for specific pathogen-free (SPF) Swine Breeding and Management of the Harbin Veterinary Research Institute, Chinese Academy of Agricultural Sciences. The piglets were randomly divided into nine groups (*n* = 3, 4, or 6). Each piglet in each experimental group was immunized intramuscularly with 2 mL of each virus containing 10^6.5^ TCID_50_ (rHuN4-F112, rHuN4-F112-C1a, rHuN4-F112-C1b, rHuN4-F112-C27, rCH-1R, rCH-1R-H1a, rCH-1R-H1b, or rCH-1R-H27). Each piglet in the control group was mock-inoculated with the same dose of MARC-145 cell culture supernatant. Blood was collected from each animal at 0, 1, 2, 3, 4, 5, 6, 7, 8, 9, 10 and 11 weeks post-inoculation, and the sera were individually prepared and stored at −80 °C.

### Serological analysis

The PRRSV-specific antibody levels in the sera were determined using a commercial IDEXX PRRS X3 ELISA Kit (IDEXX Laboratories, Westbrook, ME, USA). According to the manufacturer’s directions, samples with sample value/positive value (S/P) ratios ≥0.4 were considered positive for antibodies against PRRSV. To estimate the ability of the different rescued viruses in antibody induction in piglets, the S/*P* values of the serum samples collected from the piglets in the same group in different time points were accumulated and then the accumulated values were divided by the number of piglets in the group to obtain a value at last. The values can reflect the ability of the rescued viruses to induce antibodies in piglets to some extent.

### Neutralization analysis

The sera neutralization assay was performed as previously described [29]. First, all tested sera were heat inactivated for 30 min at 56 °C prior to testing. Each serum sample was diluted using a two-fold serial dilution technique in DMEM. Then, 100 μL of each diluted sample was mixed with an equal volume of each virus (10^3^ TCID_50_/mL). Finally, the mixtures were incubated for 1 h at 37 °C and inoculated onto MARC-145 cell monolayers prepared in 96-well plates 24 h earlier. Each diluted sample was run in four parallel repeats in 96-well plates. Thereafter, the cells were incubated at 37 °C and monitored daily for CPE. The presence of virus-specific CPE in each well was recorded after 5 days of incubation. The NA titer or cross NA titer of each serum sample against the different rescued PRRSVs was calculated using the Reed-Muench method [36]. The neutralization tests of each serum sample were repeated three times independently. The results represent the average of the duplicates. Similar to the previous description, to estimate the ability of the different rescued viruses in NA induction in piglets, the NA titers of the serum samples collected from the piglets in the same group in different time points were also accumulated and the accumulated values were also divided by the number of piglets in the group to obtain a value at last. And the values can also reflect the ability of the rescued viruses to induce NAs in piglets to some extent.

### Viremia analysis

The viremia analysis was conducted using a virus isolation assay as previously described [24]. Briefly, the sera were diluted 10-fold with DMEM and transferred to MARC-145 cell monolayers prepared in 96-well plates 24 h earlier. Then, the cells were incubated at 37 °C for 3–5 days and monitored daily for CPE. All of the samples were tested three times independently.

### Statistical analysis

The Student’s t-test was used to estimate the differences among the growth kinetics of the different rescued viruses, anti-N protein antibody and NA levels of the different rescued virus inoculated groups and cross NA titers of the anti-rHuN4-F112 sera against the different rescued viruses. Differences were considered significant at a *P* value <0.05 and extremely significant at values of *P* < 0.01 and *P* < 0.001.

## Results

### Recovery of the chimeric and parental viruses

The two rescued parental viruses were named rHuN4-F112 and rCH-1R (Fig. 1). Six chimeric viruses were successfully rescued from the chimeric infectious clones constructed by swapping the 5’UTR + ORF1a, ORF1b or ORF2–7 + 3’UTR regions between the pHuN4-F112 and pCH-1R plasmids and were individually designated rHuN4-F112-C1a, rHuN4-F112-C1b, rHuN4-F112-C27, rCH-1R-H1a, rCH-1R-H1b and rCH-1R-H27 (Fig. 1).

**Fig. 2:**
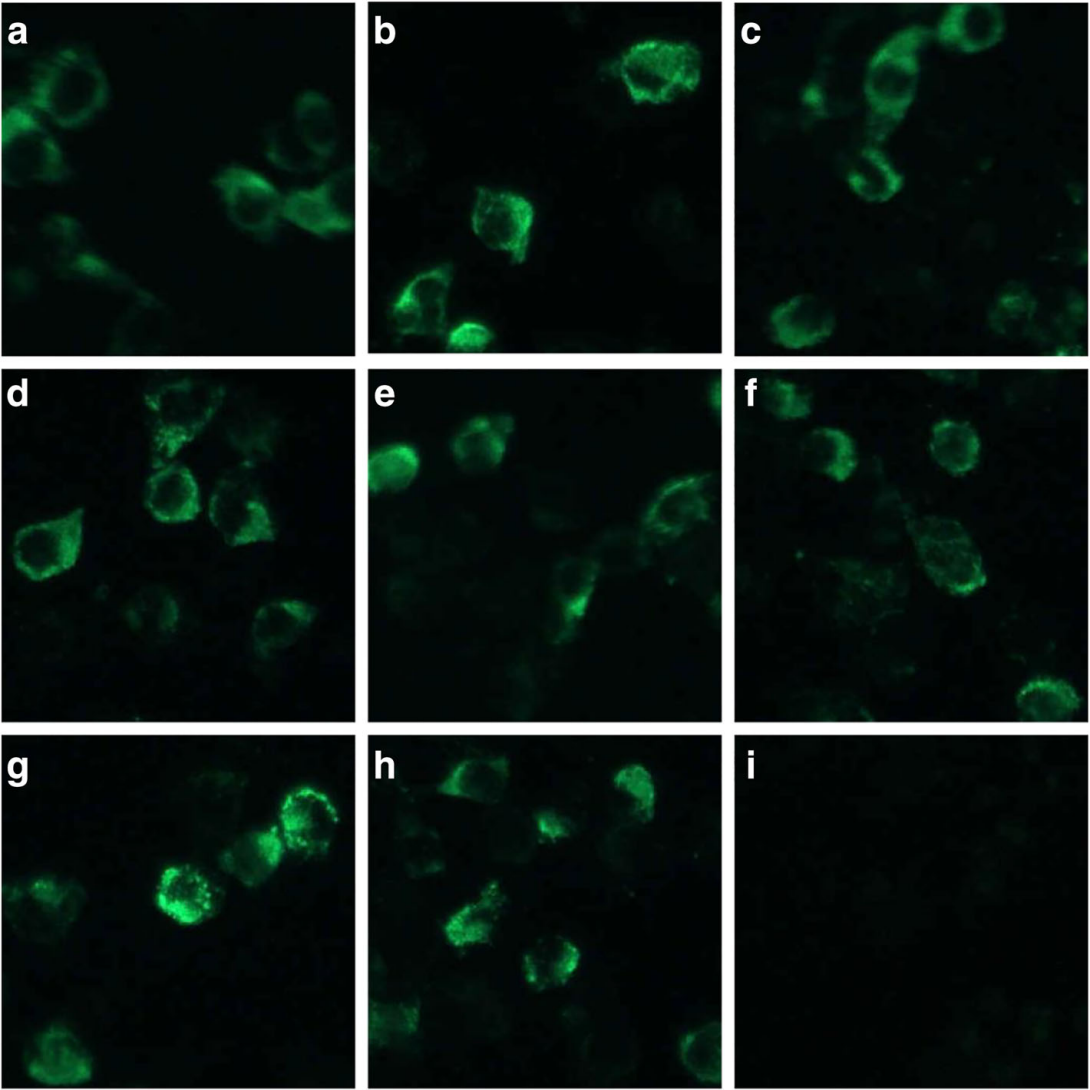
Identification of the rescued viruses with the anti-PRRSV monoclonal antibody by IFA. **a**-**h** Reactivity of the anti-PRRSV M protein monoclonal antibody against rHuN4-F112, rCH-1R, rHuN4-F112-C1a, rHuN4-F112-C1b, rHuN4-F112-C27, rCH-1R-H1a, rCH-1R-H1b and rCH-1R-H27 in MARC-145 cells, respectively. **i**: Uninfected negative control MARC-145 cells. Magnification, 400 ×

**Fig. 3:**
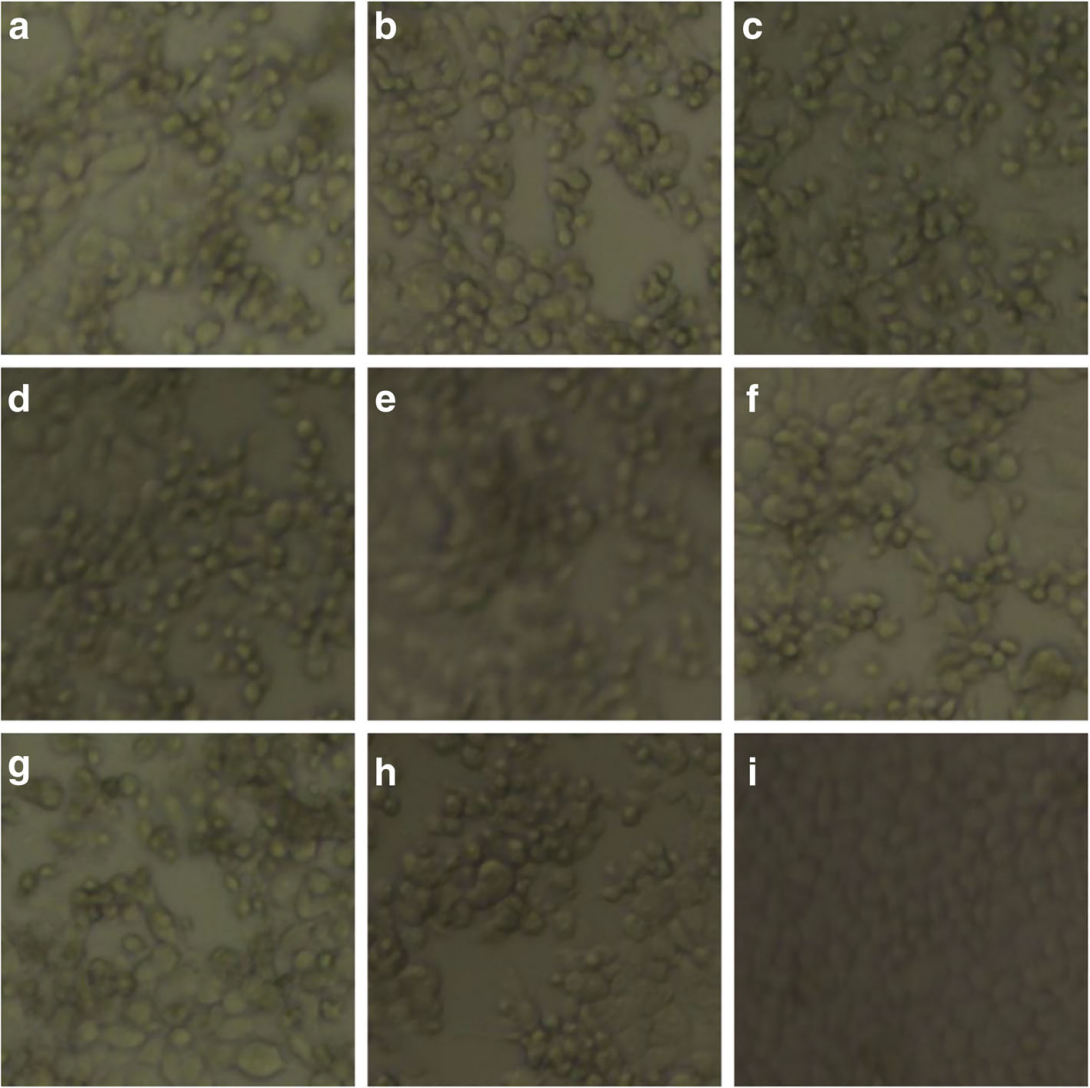
Identification of the rescued virus based on CPE. **a**-**h** Infection of MARC-145 cells with rHuN4-F112, rCH-1R, rHuN4-F112-C1a, rHuN4-F112-C1b, rHuN4-F112-C27, rCH-1R-H1a, rCH-1R-H1b and rCH-1R-H27, respectively. **i** Uninfected negative control MARC-145 cells. Magnification, 400 ×

**Fig. 4:**
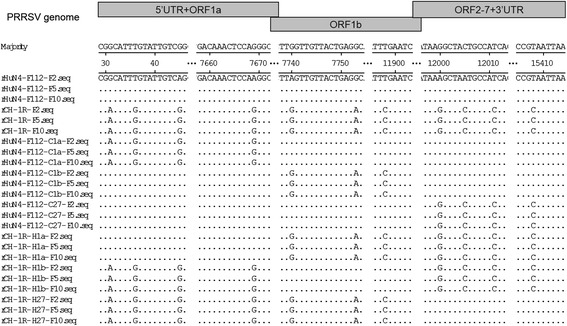
Genome sequence results of the rescued viruses of generation 2, 5 and 10. rHuN4-F112-F2, rHuN4-F112-F5 and rHuN4-F112-F10 mean that rHuN4-F112 was passaged on MARC-145 cells for 2, 5 and 10 generations, respectively. Others by this analogy

The MARC-145 cells infected with each rescued virus were positive for PRRSV based on CPE (Fig. 2) and the IFA results (Fig. 3). Furthermore, the sequencing results confirmed that the replaced regions and their flanking areas in the second-passage rescued viruses were consistent with the original design and that no additional mutations were introduced during construction (Fig. 4). No genetic variability was observed between the fifth and tenth passages of the rescued viruses (Fig. 4).

### Growth kinetics of the rescued viruses

**Fig. 5:**
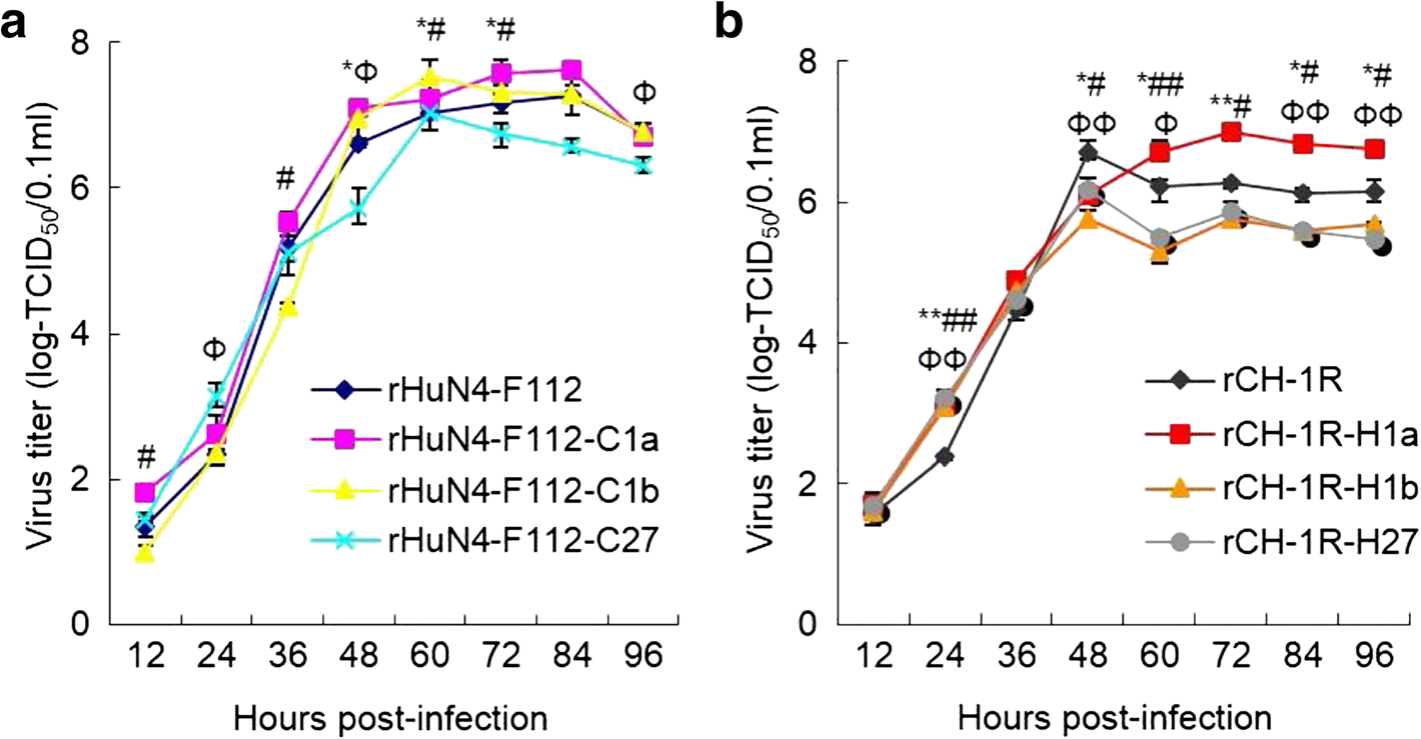
Growth kinetics of the rescued viruses in MARC-145 cells. **a** The growth kinetics of rHuN4-F112, rHuN4-F112-C1a, rHuN4-F112-C1b and rHuN4-F112-C27 in MARC-145 cells. **b** The growth kinetics of rCH-1R, rCH-1R-H1a, rCH-1R-H1b and rCH-1R-H27 in MARC-145 cells. An asterisk (*) indicates a significant difference in the viral titers between rHuN4-F112 and rHuN4-F112-C1a or between rCH-1R and rCH-1R-H1a (*P < 0.05; **P < 0.01). The pound sign (#) indicates a significant difference between rHuN4-F112 and rHuN4-F112-C1b or between rCH-1R and rCH-1R-H1b (#*P* < 0.05; ##*P* < 0.01). Phi (Φ) indicates a significant difference between rHuN4-F112 and rHuN4-F112-C27 or between rCH-1R and rCH-1R-H27 (ΦP < 0.05; ΦΦP < 0.01). The experiments were independently repeated three times and each time point was also independently repeated three times. The results represent the average of the duplicates

The growth kinetics of the chimeric viruses in parallel with their parental viruses (rHuN4-F112 and rCH-1R) were evaluated by infecting MARC-145 cells. rHuN4-F112-C1a had higher growth kinetics than its parental backbone virus rHuN4-F112 at 48 h, 60 h and 72 h (Fig. 5a). Interestingly, rHuN4-F112-C1b showed a reduced replication efficiency compared with the backbone virus rHuN4-F112 at 12 h and 36 h and an increased replication efficiency at 60 h and 72 h; conversely, rHuN4-F112-C27 exhibited an increased replication efficiency at 24 h and a reduced replication efficiency at 48 h and 96 h (Fig. 5a). Compared with the backbone virus rCH-1R, rCH-1R-H1a showed an increased replication efficiency at most time points, whereas rCH-1R-H1b and rCH-1R-H27 had reduced replication efficiencies at the same time points (Fig. 5b). These results indicated that exchanging the 5’UTR + ORF1a, ORF1b or ORF2–7 + 3’UTR affected the replication efficiencies of rHuN4-F112 and rCH-1R.

### Antibody kinetics in the sera of piglets inoculated with the rescued viruses

**Fig. 6:**
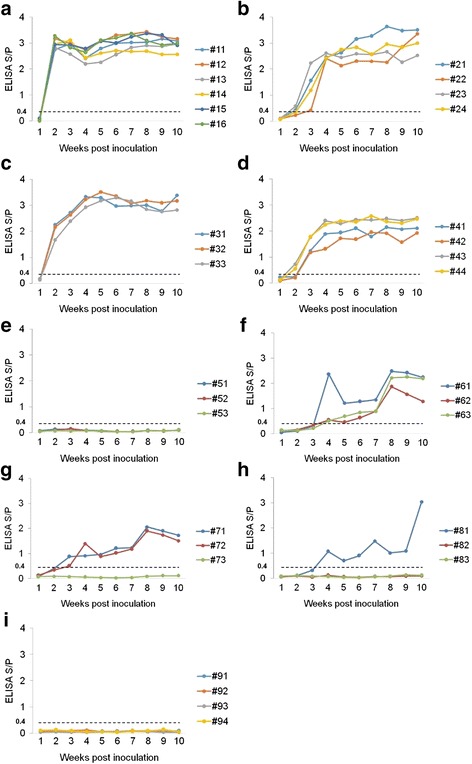
Antibody kinetics in the sera of piglets inoculated with the rescued viruses. **a**-**i** The antibody kinetics of piglets inoculated with rHuN4-F112, rHuN4-F112-C1a, rHuN4-F112-C1b, rHuN4-F112-C27, rCH-1R, rCH-1R-H1a, rCH-1R-H1b, or rCH-1R-H27 or the MARC-145 cell culture supernatant, respectively. Antibodies specific for PRRSV were detected using an IDEXX PRRS X3 ELISA kit, and the antibody level was expressed as a sample value/positive value (S/P) ratio. A ratio≥0.4 was regarded as seroconversion

Specific antibodies against the PRRSV N protein in the sera of the inoculated piglets were measured using a commercial PRRS X3 IDEXX ELISA Kit (Fig. 6). All of the piglets in the rHuN4-F112- and rHuN4-F112-C1b-inoculated groups seroconverted, whereas only half of the piglets in the rHuN4-F112-C1a- or rHuN4-F112-C27-inoculated groups had seroconverted at 2 weeks post-inoculation (Fig. 6a-6d). Conversely, the piglets in the rCH-1R-inoculated group remained seronegative throughout experiment. The piglets in the rCH-1R-H1a-, rCH-1R-H1b- and rCH-1R-H27-inoculated groups seroconverted at 4 weeks post-inoculation, and the rCH-1R-H1b- and rCH-1R-H27-inoculated groups each had one or two piglets that remained seronegative until the end of the experiment (Fig. 6e-6h). The control group remained seronegative for the duration of the experiment (Fig. 6i).

### NA kinetics in the sera of piglets inoculated with the rescued viruses

**Fig. 7:**
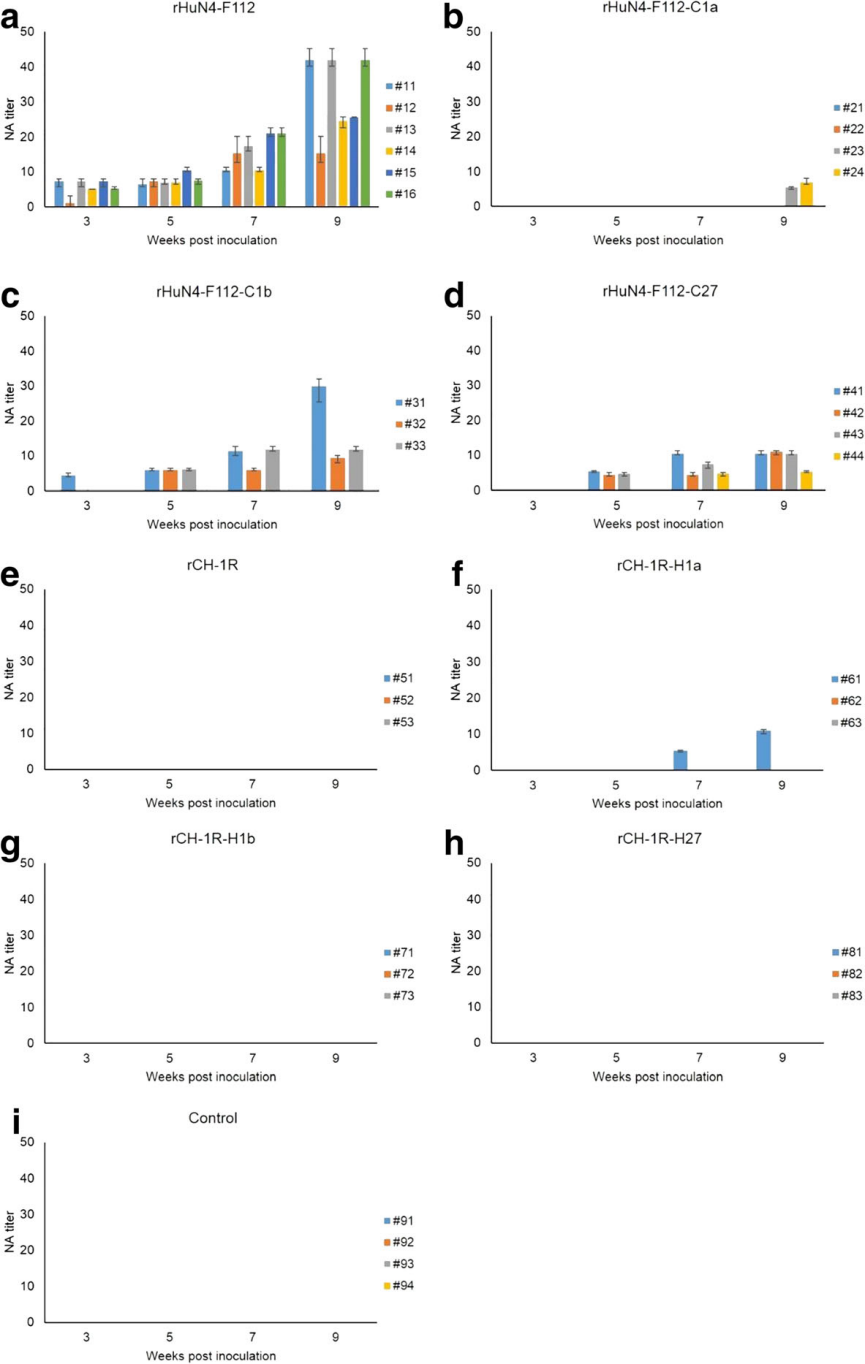
Neutralizing antibody (NA) kinetics in the sera of piglets inoculated with the rescued viruses. **a**-**i** The NA kinetics of piglets inoculated with rHuN4-F112, rHuN4-F112-C1a, rHuN4-F112-C1b, rHuN4-F112-C27, rCH-1R, rCH-1R-H1a, rCH-1R-H1b, or rCH-1R-H27 or the MARC-145 cell culture supernatant, respectively. The NA titers of the experimental group serum samples against the inoculated rescued virus strains were calculated. The NA titers of the control group serum samples against rHuN4-F112 and rCH-1R were also calculated. The neutralization tests of each serum sample were repeated three times independently. The results represent the average of the duplicates

The anti-PRRSV NAs in the sera of the inoculated piglets were measured using the neutralization test. As shown in Fig. 7, NAs were generated as soon as 3 weeks post-inoculation in the rHuN4-F112- and rHuN4-F112-C1b-inoculated groups, and the highest NA titer was greater than 45 (Fig. 7a). In contrast, the NAs of the rHuN4-F112-C1a-inoculated group were generated 9 weeks post-inoculation, and the highest NA titer was less than 10 (Fig. 7b). Additionally, the NAs of the rHuN4-F112-C27-inoculated group were generated 4 weeks post-inoculation, and the highest NA titer was approximately 10 (Fig. 7d). Throughout the experiment, no piglets in the rCH-1R-, rCH-1R-H1b- and rCH-1R-H27-inoculated groups produced NAs (Fig. 7e, g, h). However, one piglet in the rCH-1R-H1a-inoculated group generated NAs at 7 weeks post-inoculation (Fig. 7f). The control group piglets produced no NAs against rHuN4-F112 or rCH-1R during the experiment (Fig. 7i).

### Ability of the rescued viruses in antibody and NA induction in piglets

**Fig. 8:**
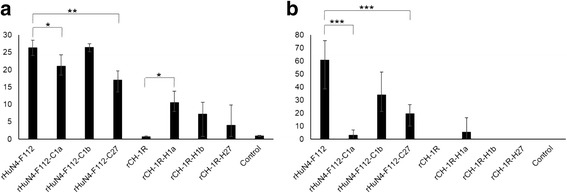
Values to reflect the ability of the rescued viruses in antibody induction in piglets. **a**-**b** The values to reflect the ability of the rescued viruses in anti-N protein antibody and NA induction in piglets, respectively. The values were calculated by accumulating the S/P ratios or NA titers of the serum samples collected from the piglets in the same group in different time points and dividing by the number of piglets in the group. An asterisk (*) indicates a significant difference of the ability in anti-N antibody or NA induction in piglets between the two different rescued viruses (*P < 0.05; **P < 0.01; ***P < 0.001)

The ability of the rescued viruses in anti-N antibody and NA induction in piglets were analyzed (Fig. 8). Results indicated that the ability of the rHuN4-F112 in anti-N antibody or NA induction in piglets was significantly better than that of rHuN4-F112-C1a or rHuN4-F112-C27, whereas the difference between rHuN4-F112 and rHuN4-F112-C1b was not obvious (Fig. 8). Correspondingly, we found that the ability of the rCH-1R in anti-N antibody induction in piglets was significantly worse than that of rCH-1R-H1a, whereas the difference between rCH-1R and rCH-1R-H1b or rCH-1R-H27 was not obvious (Fig. 8a). However, there was no significant difference of the ability in NA induction between rCH-1R and rCH-1R-1a, rCH-1R-1b or rCH-1R-27 (Fig. 8b).

### Cross NA titers of the rHuN4-F112-inoculated piglet sera against the different rescued viruses

**Fig. 9:**
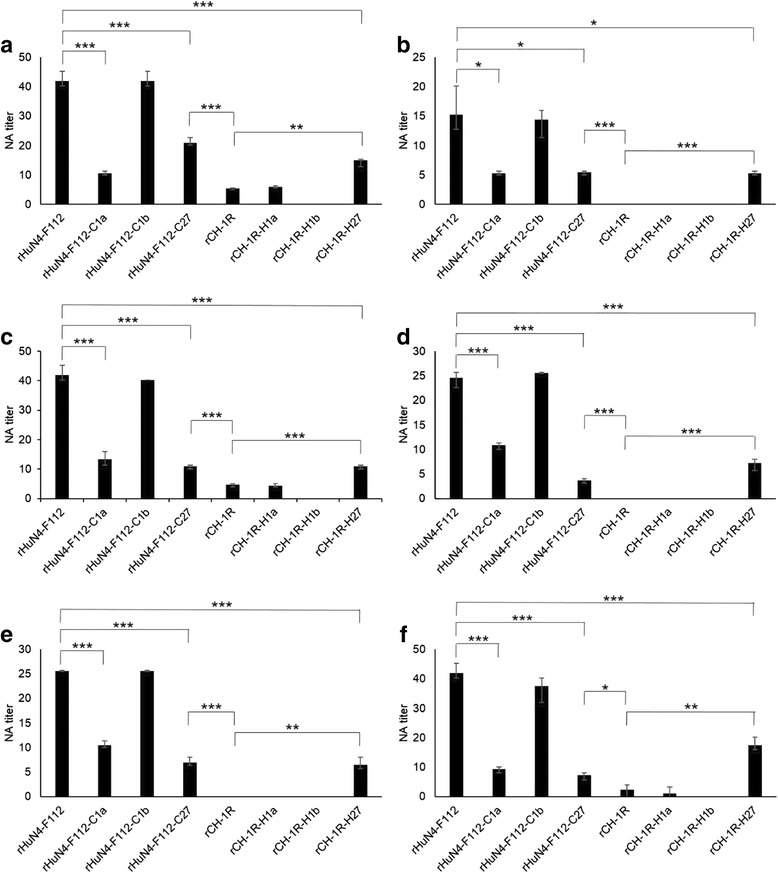
Cross neutralizing antibody (NA) titers of the rHuN4-F112-inoculated piglet sera against different rescued viruses. **a**-**f** The NA titers or cross NA titers of rHuN4-F112-inoculated piglet sera (#11, #12, #13, #14, #15 and #16) against rHuN4-F112, rHuN4-F112-C1a, rHuN4-F112-C1b, rHuN4-F112-C27, rCH-1R, rCH-1R-H1a, rCH-1R-H1b and rCH-1R-H27. All the serum samples were collected at 9 weeks post-inoculation with rHuN4-F112. An asterisk (*) indicates a significant difference in the NA titers between two different rescued viruses (*P < 0.05; **P < 0.01; ***P < 0.001). The neutralization tests of each serum sample were repeated three times independently. The results represent the average of the duplicates

The NA titers or cross NA titers of the rHuN4-F112-inoculated piglet sera (#11, #12, #13, #14, #15 and #16) against rHuN4-F112, rHuN4-F112-C1a, rHuN4-F112-C1b, rHuN4-F112-C27, rCH-1R, rCH-1R-H1a, rCH-1R-H1b and rCH-1R-H27 were measured using the neutralization test (Fig. 9). All six serum samples were collected 9 weeks post-inoculation with rHuN4-F112. The results indicated that there was no significant difference between the NA titers against rHuN4-F112 and rHuN4-F112-C1b in the rHuN4-F112-inoculated piglet sera. However, the NA titers against rHuN4-F112 were significantly higher than the NA titers against rHuN4-F112-C1a, rHuN4-F112-C27 and rCH-1R-H27 in the rHuN4-F112-inoculated piglet sera. In contrast, the NA titers against rCH-1R were significantly lower than the NA titers against rCH-1R-H27 and rHuN4-F112-C27 in the rHuN4-F112-inoculated piglet sera. Moreover, no significant difference was found between the NA titers against rCH-1R and rCH-1R-H1a in the rHuN4-F112-inoculated piglet sera.

### Viremia in the piglets inoculated with the rescued viruses

The viremia of the piglets inoculated with the rescued viruses was examined using the virus isolation assay (Table 1). For the rHuN4-F112-inoculated group, virus was isolated from all serum samples in the first two weeks post-inoculation and half of the serum samples in the third week post-inoculation. However, for the rHuN4-F112-C1a-, rHuN4-F112-C1b-, and rHuN4-F112-C27-inoculated groups, the virus was isolated from only a few serum samples. In contrast, the virus was not isolated in any serum sample from the rCH-1R-inoculated group. However, virus was isolated from three serum samples and one serum sample from the rCH-1R-H1a-inoculated and rCH-1R-H1b-inoculated groups, respectively. Additionally, virus was not isolated from any of the serum samples from the rCH-1R-H27-inoculated and control groups.

**Table 1:** Virus isolation results from the serum samples of piglets inoculated with the rescued viruses

Groups	Pig no.	Weeks post-inoculation										
		0	1	2	3	4	5	6	7	8	9	10
rHuN4-F112 inoculated group	#11	-^a^	+^b^	+	+	−	−	−	−	−	−	−
	#12	−	+	+	−	−	−	−	−	−	−	−
	#13	−	+	+	+	−	−	−	−	−	−	−
	#14	−	+	+	−	−	−	−	−	−	−	−
	#15	−	+	+	−	−	−	−	−	−	−	−
	#16	−	+	+	+	−	−	−	−	−	−	−
rHuN4-F112-C1a inoculated group	#21	−	−	−	−	−	+	−	−	−	−	−
	#22	−	−	−	−	−	−	−	−	−	−	−
	#23	−	−	−	−	−	−	−	−	−	−	−
	#24	−	−	−	−	+	−	−	−	−	−	−
rHuN4-F112-C1b inoculated group	#31	−	−	−	+	−	−	−	−	−	−	−
	#32	−	−	−	−	−	−	−	−	−	−	−
	#33	−	−	−	−	−	−	−	−	−	−	−
rHuN4-F112-C27 inoculated group	#41	−	−	−	−	−	−	−	−	−	−	−
	#42	−	−	+	−	−	−	−	−	−	−	−
	#43	−	−	−	−	−	−	−	−	−	−	−
	#44	−	−	−	−	−	−	−	−	−	−	−
rCH-1R inoculated group	#51	−	−	−	−	−	−	−	−	−	−	−
	#52	−	−	−	−	−	−	−	−	−	−	−
	#53	−	−	−	−	−	−	−	−	−	−	−
rCH-1R-H1a inoculated group	#61	−	−	−	+	+	+	−	−	−	−	−
	#62	−	−	−	−	−	−	−	−	−	−	−
	#63	−	−	−	−	−	−	−	−	−	−	−
rCH-1R-H1b inoculated group	#71	−	−	−	−	−	−	−	−	−	−	−
	#72	−	−	−	+	−	−	−	−	−	−	−
	#73	−	−	−	−	−	−	−	−	−	−	−
rCH-1R-H27 inoculated group	#81	−	−	−	−	−	−	−	−	−	−	−
	#82	−	−	−	−	−	−	−	−	−	−	−
	#83	−	−	−	−	−	−	−	−	−	−	−
Control group	#91	−	−	−	−	−	−	−	−	−	−	−
	#92	−	−	−	−	−	−	−	−	−	−	−
	#93	−	−	−	−	−	−	−	−	−	−	−
	#94	−	−	−	−	−	−	−	−	−	−	−

^a^ “-” indicates that the virus was not isolated in the serum sample

^b^ “+” indicates that the virus was isolated in the serum sample

## Discussion

PRRSV continues to be one of the leading swine pathogens and causes great economic losses for the swine industry worldwide [1, 2, 24]. Our previous study found that the HP-PRRSV vaccine strain HuN4-F112 induced significantly higher NA titers than the classical PRRSV vaccine strain CH-1R [29]. That study confirmed that the B epitope, which is generally believed to be the major neutralizing epitope of PRRSV, was not a neutralizing epitope in HP-PRRSV [29]. Based on the significant differences in the NA titers induced by HuN4-F112 and CH-1R, we explored the generation of anti-PRRSV NAs and tried to determine the region(s) of the virus genome that contributed to neutralization of HP-PRRSV.

In the present study, large fragments of the genome were initially swapped between rHuN4-F112 and rCH-1R using a reverse genetic procedure to analyze the possible contributor to neutralization. The six chimeric viruses (rHuN4-F112-C1a, rHuN4-F112-C1b, rHuN4-F112-C27, rCH-1R-H1a, rCH-1R-H1b and rCH-1R-H27) were individually constructed, rescued and identified (Fig.1-4). The growth kinetics of the chimeric viruses in parallel with of the growth kinetics of their parental viruses (rHuN4-F112 or rCH-1R) were evaluated by infecting MARC-145 cells. Exchanging any one of the 5’UTR + ORF1a, ORF1b or ORF2–7 + 3’UTR regions could affect the replication efficiencies of rHuN4-F112 and rCH-1R (Fig. 5).

The anti-N protein antibody levels of the eight rescued PRRSVs used to infect piglets were determined by ELISA (Fig. 6). The data indicated that the seroconversion times of the rHuN4-F112- and rHuN4-F112-C1b-inoculated groups were shorter than the seroconversion times of the rHuN4-F112-C1a- and rHuN4-F112-C27-inoculated groups, although the latter two groups also produced high levels of antibodies (S/*P* ≥ 2.0) (Fig. 6). The replacement of 5’UTR + ORF1a or ORF2–7 + 3’UTR in rHuN4-F112 with the corresponding region from rCH-1R greatly decreased the anti-N protein antibody responses in the rHuN4-F112-infected piglets, whereas no difference was observed in the anti-N protein antibody responses following replacement of ORF1b of rHuN4-F112 with the corresponding region from rCH-1R (Fig. 8a). Additionally, most piglets in the rCH-1R-H1a-, rCH-1R-H1b- and rCH-1R-H27-inoculated groups seroconverted at 4 weeks post-inoculation, whereas the rCH-1R-inoculated groups remained seronegative for the duration of the experiment (Fig. 6). The replacement of 5’UTR + ORF1a, ORF1b and ORF2–7 + 3’UTR in rCH-1R with the corresponding regions in rHuN4-F112 increased the anti-N protein antibody responses (Fig. 6). However, we can find the significant difference in anti-N antibody responses between rCH-1R and rCH-1R-H1a, whereas there was no significant difference between rCH-1R and rCH-1R-H1b or rCH-1R-H27 (Fig. 8a).

The NA titers of the eight rescued PRRSVs used to infect piglets were also determined by the virus neutralization test (Fig. 7). The results were similar to the production of anti-N protein antibodies described above. The appearance times of the NAs were shorter in the rHuN4-F112- and rHuN4-F112-C1b-inoculated groups than in the rHuN4-F112-C1a- and rHuN4-F112-C27-inoculated groups (Fig. 7). Furthermore, the NA titers were higher in the rHuN4-F112- and rHuN4-F112-C1b-inoculated groups than in the rHuN4-F112-C1a- and rHuN4-F112-C27-inoculated groups (Fig. 7). Further analysis showed that the ability of the rHuN4-F112 in NA induction in piglets was significantly better than that of rHuN4-F112-C1a or rHuN4-F112-C27, whereas the difference between rHuN4-F112 and rHuN4-F112-C1b was not obvious (Fig. 8b). Thus, replacement of the 5’UTR + ORF1a or ORF2–7 + 3’UTR of rHuN4-F112 with the corresponding region in rCH-1R greatly delayed the appearance of NAs and decreased the NA titers in the rHuN4-F112-infected piglets, whereas no difference was observed in the NA responses when ORF1b of rHuN4-F112 was replaced with the corresponding region from rCH-1R. Additionally, no piglets produced NAs in the rCH-1R-, rCH-1R-H1b- and rCH-1R-H27-inoculated groups, and one piglet in the rCH-1R-H1a-inoculated group generated NAs at 7 weeks post-inoculation (Fig. 7). These data indicated that replacement of 5’UTR + ORF1a in rCH-1R with the corresponding region from rHuN4-F112 increased the NA responses. However, the differences of the ability in NA induction between rCH-1R and rCH-1R-1a, rCH-1R-1b or rCH-1R-27 was not obvious (Fig. 8b).

Based on the above results, we found that the 5’UTR + ORF1a and ORF2–7 + 3’UTR regions of PRRSV was closely related to the production of anti-N protein antibodies and NAs. As we all know, the 5′ and 3’UTRs of PRRSV may be involved in replicative and translational functionality, although the exact functions are poorly understood [37]. The NSPs encoded by ORF1a assemble into a membrane-associated enzyme complex that directs viral replication and transcription; however, the internal functions and associated mechanisms of this interaction are also unknown [38]. In addition, the nucleocapsid N protein of PRRSV builds the scaffold for the genomic RNA, and several membrane proteins are incorporated into the viral envelope [39]. GP2/3/4 forms a disulfide-linked heterotrimeric complex in virus particles that is required for virus entry and governs cell tropism [39, 40]. GP5 is disulfide-linked to M, and the heterodimer is required for virus budding [40]. In the present study, the replacements of the 5’UTR + ORF1a or ORF2–7 + 3’UTR region between rHuN4-F112 and rCH-1R may change the structure or function of these regions, alter the characteristics of the viruses further, and affect the replication and antibody responses of the viruses in piglets at last.

PRRSV NAs are directed against the surface proteins, including GP2, GP3, GP4, GP5 and M [41–47]. GP5 was favored as the most relevant protein [30, 31, 48–51]. In the present study, we found that the NA titers in the rHuN4-F112-inoculated piglet sera against rHuN4-F112-C27 were significantly lower than the NA titers against rHuN4-F112 itself (Fig. 9). Correspondingly, the NA titers from rHuN4-F112-inoculated piglet sera against rCH-1R-H27 were significantly higher than the NA titers against rCH-1R (Fig. 9). These results suggested that ORF2–7 was the neutralization region of PRRSV, which is consistent with previous reports. Additionally, no significant difference was found between the NA titers against rHuN4-F112 and the NA titers against rHuN4-F112-C1b or between the NA titers against rCH-1R and the NA titers against rCH-1R-H1b in the sera from the rHuN4-F112-inoculated piglets (Fig. 9). This result indicated that ORF1b was not the neutralization region of PRRSV. Surprisingly, the NA titers from the rHuN4-F112-inoculated piglet sera against rHuN4-F112-C1a were significantly lower than the NA titers against rHuN4-F112 itself. Conversely, the NA titers of the rHuN4-F112-inoculated piglet sera against rHuN4-F112-C27 were significantly higher than the NA titers against rCH-1R (Fig. 9). These results indicated that ORF1a was also a neutralization region of PRRSV. Most researchers believe that the neutralization region of PRRSV is located in ORF2–6, and many genetically engineered vaccines have been designed based on this region [52–56]. At present, these vaccines are still in the experimental stage, possibly because the neutralization region remains unclear. Recently, it was described that NSP2 was a virion-associated structural PRRSV protein that existed in or on viral particles in multiple isoforms [57]. Further study demonstrated that NSP2 integrated into membranes with an unexpected topology in which the amino (N)-terminal (cytoplasmic) and C-terminal (luminal) domains were orientated on opposite sides of the membrane surface [58]. Taken together, these reports support our results showing that the neutralization region of ORF1a may be located at the NSP2 region. Of course, this issue requires further study.

The virus isolation results showed that rHuN4-F112 induced viremia in the first three weeks, whereas rCH-1R did not induce viremia during the experiment. The chimeric viruses rHuN4-F112-C1a, rHuN4-F112-C1b, rHuN4-F112-C27, rCH-1R-H1a and rCH-1R-H1b induced viremia only during limited time points. Moreover, rCH-1R-H27 did not induce viremia during the experiment. These results indicated that exchanging 5’UTR + ORF1a, ORF1b or ORF2–7 + 3’UTR affected the replication efficiencies of rHuN4-F112 and rCH-1R in vivo. Generally, a viremia that is higher and has a longer duration indicates a stronger replication ability of PRRSV in piglets and thus results in higher antibody production. We found consistent results in the rHuN4-F112 and rCH-1R inoculated groups. However, we did not find complete consistency in the other chimeric virus inoculation groups (Figs. 6-7; Table 1). This discrepancy was very confusing, and the internal mechanism requires further study.

In the present study, we found that rCH-1R strain could not induce antibody response and viremia in piglets (Figs. 6-7; Table 1). It seems that CH-1R can not grow in pigs. However, if given more immunization dosages or vaccination times of CH-1R, the pigs may produce low levels of immune response and viremia. Recently, our colleagues reported that CH-1R can induce lower levels of immune responses and viremia compared to HuN4-F112 and supply partial protection to the heterologous PRRSV challenge [28]. To date, the protective mechanism of CH-1R vaccine is unclear and future investigation is needed.

## Conclusion

The present study constructed and rescued 6 chimeric viruses by exchanging 5’UTR + ORF1a, ORF1b, and ORF2–7 + 3’UTR between rHuN4-F112 and rCH-1R. All of these fragments affected the replication efficiencies of rHuN4-F112 and rCH-1R in vitro. Additionally, both 5’UTR + ORF1a and ORF2–7 + 3’UTR affected the anti-N antibody and NA responses to rHuN4-F112 and rCH-1R in piglets. Furthermore, we confirmed for the first time that ORF1a contains a neutralization region. This study provides important information for further study of the generation of anti-PRRSV NAs.

## Abbreviations

CPE: Cytopathic effects
DMEM: Dulbecco’s modified Eagle’s medium
EU-type: European type
FBS: Fetal bovine serum
h: hours
HP-PRRSV: Highly pathogenic porcine reproductive and respiratory syndrome virus
IFA: Indirect immunofluorescence assay
LV: Lelystad virus
MOI: Multiplicity of infection
NA: Neutralizing antibody
NA-type: North American type
NSP: Non-structural protein
ORF: Open reading frame
PRRS: Porcine reproductive and respiratory syndrome
PRRSV: Porcine reproductive and respiratory syndrome virus
SPF: Specific pathogen-free
TCID_50_/mL: 50% tissue culture infective doses per mL
UTR: Untranslated region
